# Artificial intelligence in contact sports research: Promise and peril for predicting and preventing brain injury

**DOI:** 10.1113/EP093659

**Published:** 2026-05-05

**Authors:** Danny W. Walmsley, Damian M. Bailey

**Affiliations:** ^1^ Neurovascular Research Laboratory, Faculty of Life Sciences and Education University of South Wales Pontypridd UK; ^2^ Bexorg Inc New Haven Connecticut USA

Contact sports, including rugby, American football and martial arts, entail an inherent and recognised risk of brain damage, both acute and cumulative. These provide a unique opportunity to approach and understand accelerated cognitive decline and its underpinning mechanisms.

Concussion is a form of mild traumatic brain injury (mTBI); an acceleration of the head which alters mental status and commonly causes, amongst other potential symptoms, nausea, vomiting, headache and transient cognitive impairment (Patricios et al., 2023). The acute symptoms typically present within hours of the impact and reverse within weeks. However, post‐concussion syndrome is experienced by ∼6% of concussed individuals, whereby symptoms continue or are triggered by physical or cognitive load for several months post‐injury (Woo et al., 2021). Concussion and history of recurrent contact have previously been linked to impaired cerebral and systemic vascular function and accelerated cognitive decline subsequent to a systemic elevation in free radical‐mediated oxidative–inflammatory–nitrosative stress (Owens et al., 2021, 2023) which, when exaggerated, is known to cause structural damage to the neurovascular unit (Bailey et al., 2022).

Despite recent advances in diagnostic technologies (from MRI‐based approaches to accelerometer‐equipped gumshields), the identification and management of concussion remain heavily reliant on subjective symptom reporting and nascent, yet promising, biomarker assessments (Janigro et al., 2020). However, neurological research currently stands on the precipice of significant change, with rapidly advancing imaging technologies and shotgun multi‐omics analyses generating volumes of raw data that need to be deciphered ‘intelligently’.

To that end, artificial intelligence (AI) offers new opportunities to improve how we detect, monitor, treat, and ultimately prevent sports‐related (SR)‐mTBI. However, such Promethean promises of AI are not without their downsides. AI carries a unique set of threats: a Pandora's box of potential dangers that challenge scientific rigour, research transparency and intellectual culture.

## 1 CONCEPTUAL DOMAINS OF AI

In their recent *Nature* Viewpoint, Messeri & Crockett (2024) outlined four overarching responsibilities that AI bears, including acting as (1) an oracle, whereby large language models (LLMs) have the capability to digest and synthesize large corpora of relevant written text, ideal for hypothesis generation and knowledge gap identification; (2) a surrogate, with the possibility of AI ‘agents’ complementing or replacing human‐collected data; (3) a quant, with the capacity to handle large, high dimensional data sets and detect subtle, multi‐faceted patterns; and (4) an arbiter, imagined for use by journal editors and reviewers to address the burgeoning increases in manuscript submissions by ‘screening’ for quality and weeding out paper mill manuscripts. We may imagine these roles filled in SR‐mTBI research as follows.

Oracles in this field include LLMs trained on medical information and relevant, reliable and reviewed research (e.g. open evidence) which can summarise large volumes of text to save time in the literature review and study the conception and hypothesis generation stages of research (Wagner et al., 2022), provided researchers remain vigilant against the risk of ‘hallucinations’ (see below).

Surrogates for human patients/participants may soon become viable, whereby AI agents complement or stand in for human‐generated data. In contact sports, we may utilise more sophisticated simulated head and neck models to predict the most affected brain structures (Zhan et al., 2026) in impacts of varying magnitudes and directions, in turn guiding medical treatment and rehabilitation.

Artificially intelligent programs, even language models, are ultimately mathematical models, making their use as ‘quants’ very intuitive. To apply this in the domain of sport, AI‐based quantitative approaches could analyse sensor data from helmets and instrumented gumshields to quantify not only the magnitude of linear and rotational accelerations, but also the location, direction and point of application of impact, together with athlete‐specific modifiers such as anthropometry, genetics, neck strength, neuromuscular control and prior concussion history. Incorporating these variables allows models to account for marked inter‐individual variability in susceptibility to brain injury, acknowledging that concussion risk depends not solely on impact magnitude, but on the interaction between impact biomechanics, impact location, and the individual's structural and physiological tolerance to mechanical loading. Integration of these factors may permit more accurate, personalised prediction of brain regions at risk and the likely severity of injury, rather than reliance on force metrics alone (Antrobus et al., 2021; Reneker et al., 2019; Zhan et al., 2026).

Though Messeri & Crockett (2024) imagined the arbiter as a quality screening agent for journals, which now receive overwhelming volumes of submissions, one can also envision an independent AI arbiter in sporting contexts to make return‐to‐play decisions for athletes. By synthesising both subjective symptom reporting and psychological readiness with objective physiological and fluid‐borne biomarker data (e.g. UCH‐L1, GFAP and S100B; Salgado et al., 2026), such tools should support, rather than replace, clinical judgement in return‐to‐play decisions. Recovery from concussion is not defined solely by physiological normalisation, but also by the athlete's perceived readiness, confidence, and tolerance to re‐exposure to contact, all of which influence the risk of subsequent injury. Integrative approaches may therefore assist independent medical professionals in making more informed decisions, while reducing the influence of competitive or organisational pressures to return prematurely. Objective evidence of recovery may also help build player confidence in return‐to‐play decisions, provided these metrics are interpreted within a comprehensive clinical framework and not used in isolation. Alternatively, to streamline the administrative burden of research, such AI arbiters may be used by internal ethics committees to pre‐screen applications and ensure they align with established safety and ethical guidelines, prior to review by human committees.

## 2 AGENTIC DISCOVERY: UTILISING AI‐DESIGNED PROTOCOLS AND SELF‐DRIVING LABORATORIES

AI systems devising and conducting experiments are already a reality in biology and chemistry with the advent of ‘self‐driving’ laboratories, capable of automating and accelerating the scientific method through hypothesis generation, experimental design and conduct, and data analysis, all in a repeated build–test–learn cycle (Szymanski et al., 2023; Tom et al., 2024). Such automation is not a novel concept, with, for example, researchers at Aberystwyth University having developed an autonomous ‘Robot Scientist’ which identified several key metabolic genes in a strain of yeast over 15 years ago (Sparkes et al., 2010).

We may begin to harness such automation in SR‐mTBI research not only with automated systems for conducting existing, laborious assays, but with automated scientific agents in build–test–learn–repeat loops to develop ever more sensitive assays for given biomarkers, a necessary process to produce more objective assessments for SR‐mTBI diagnosis and effective management (Wilde et al., 2022). The development of such assays for proteins, for example, is currently accelerated by the open‐access database of protein structures pioneered by AlphaFold, a deep learning AI model (Fang et al., 2025; Varadi et al., 2022).

While many key techniques are currently out of the grasp of machines, requiring trained researchers for tasks such as cannulation and ultrasonography, we can envision such build–test–learn–repeat systems for designing study protocols from reading the existing literature, generating hypotheses and analysing raw data. Such systems would undoubtedly streamline the SR‐mTBI research process.

## 3 BESPOKE AI: CUSTOM‐MADE MACHINE LEARNING

There exists a range of tools, some of which are powered by AI themselves, for developing machine learning systems. These include libraries within popular coding languages such as Python (Scikit‐learn, PyTorch, TensorFlow, etc.), R (Caret and TidyVerse), and Java (Deeplearning4j, Weka, and Apache Spark MLlib). These toolkits allow researchers to build their own machine learning (ML) models for various purposes. For example, building a language model to classify survey answers, such as in an SR‐mTBI‐symptom questionnaire, image‐recognition models to identify and measure key structures in an ultrasound/MRI image (Lui et al., 2020), or an ML model to predict athlete return‐to‐play timelines based on a multitude of data sources (Thomas & Arnett, 2025). In our laboratory, we have implemented AI‐generated code to create bespoke data analysis tools for local processing of chemical assay data, streamlining a considerably time‐consuming process.

It is important to note that when developing or using such tools outside one's primary area of expertise, appropriate verification procedures are essential. For locally run applications used within a controlled research environment, risks can be minimised provided that no identifiable participant data are transmitted to external or cloud‐based services. In these cases, validation should include systematic testing of the software, confirmation that outputs are consistent with expected behaviour, and, where possible, independent code review. For web‐based or cloud‐hosted applications, additional safeguards are required due to the increased risk of data security and privacy breaches. Any systems connected to the internet should therefore be reviewed in accordance with institutional data‐governance policies and, where appropriate, assessed by information technology or cybersecurity specialists to ensure compliance with research governance and data protection regulations.

We may also visualise ML paradigms being used in SR‐mTBI research to uncover subtle, multi‐dimensional relations, integrating independent elements of neurophysiology to unravel complex relationships between blood‐borne molecular biomarkers of oxidative–inflammatory–nitrosative stress and associated proteins that reflect structural integrity of the neurovascular unit, cerebral blood flow regulation, data from wearable devices (sleep quality tracking via actigraphy, pedometry, NEAT tracking, etc.), cognitive function, etc. Ultimately, these models could both predict clinical neurological outcomes in contact‐sports athletes and, through transparent and explainable frameworks, deepen mechanistic insight – an approach we are already pursuing in collaborative rugby brain‐health studies with the charity Head For Change. Only through such an integrated understanding could we begin to best treat and protect players. Crucially, such custom‐built applications are designed not only for laboratory use and academia but also to have real‐world applications, such as the processing of raw data (from accelerometers, biomarkers, video analyses), determining the presence of SR‐mTBIs, and informing both athletes and clinicians throughout the recovery process.

## 4 INTERPRETATION, DIAGNOSIS AND MANAGEMENT

Deep learning systems, characterised by their use of deep neural networks (DNNs) inspired by the structure of the human brain, possess unique and powerful capabilities for data interpretation. For example, raw data from helmet accelerometers can be processed by DNNs to extrapolate head kinematics, force magnitudes, and the location of the head struck during collisions (Zhan et al., 2026). These multi‐metric parameters can subsequently inform medical staff at sporting events as to the likelihood that a concussion has been sustained.

AI systems are also demonstrating proficiency in diagnosing the early‐stage effects of mTBI, as several models have been built to detect Alzheimer's disease from speech analysis (Ding et al., 2024). Such models could be imagined for SR‐mTBI diagnosis by analysing players’ speech characteristics. Indeed, beyond the initial diagnosis (perhaps performed in part by AI‐powered assessment tools), AI stands to shift the management of concussion recovery. Effective recovery protocols are not uniform but tailored to the individual, and thus the shift towards precision medicine must also manifest in the post‐mTBI recovery process, given the heterogeneity in both symptomology and timespan (Beard et al., 2024). In one such case, Rosenblatt et al. (2021) used measures including pain, depression/anxiety, cognition and sleep disturbances to accurately cluster subtypes of concussion, from ‘minimally complex’ to ‘extremely complex’, using an ML model programmed in Python. Such approaches move concussion treatment from a static, checklist‐based model to a dynamic and data‐driven framework capable of personalising rehabilitation and optimising both recovery time and athlete longevity.

## 5 POTENTIAL RISKS OF AI APPLICATION

While AI offers substantial benefits for neuroscience research, its careless application poses serious risks to scientific rigour, transparency, culture, and athlete safety. Generative AI systems pose a unique set of potential pitfalls. LLMs, for example, currently have two key issues. The first is their habitual ‘hallucinations’ in which they may state mistruths as absolute fact. The risk here is obvious: researchers using LLMs to summarise large bodies of literature into key outcomes may take hallucinations at their word, going forward through their research process with unsupported claims or, worse yet, blatant lies at the foundation of their hypotheses. Secondly, LLMs may perpetuate and, through their growing adoption, amplify existing societal biases present in their training corpus. An AI model trained on historically biased medical data could, for example, produce inequitable diagnostic or management recommendations, a risk confirmed in studies showing significant gender and racial biases in even the most advanced LLMs (Fang et al., 2024).

A further concern is that many generative systems are designed to be helpful, plausible and agreeable, which may inadvertently reinforce confirmation bias for users who seek reassurance rather than critique. One practical countermeasure is to use these tools adversarially by, for example, prompting them to identify weaknesses in study design, alternative interpretations, or reasons why a hypothesis may be incorrect. Equally, if used critically and transparently, AI may in fact enhance innovative inquiry by enabling researchers to interrogate larger and more complex datasets, generate unconventional hypotheses and test ideas more rapidly than would otherwise be possible. Fortunately, misuse is likely to be self‐limiting as researchers who over‐rely on AI without appropriate verification risk rapid loss of credibility, as the legal and ethical responsibility for the accuracy, integrity and originality of the work remains entirely with the authors.

Importantly, and the focus of Messeri & Crockett's (2024) concerns, is the risk that uncareful AI adoption in the scientific community poses to scientific integrity, culture and creativity. As the ‘publish or perish’ model incentivises voluminous academic output, shouldering the burden to generative AI systems may become commonplace. In such a case, scientific progress would lose its genuine innovative inquiry as research begins to gravitate around those questions most easily answered with AI in an echo chamber of scientific monoculture. Thus, we may begin to ‘produce more but understand less’ (Messeri & Crockett, 2024).

AI systems must be fully explainable in how they reach their conclusions. Private companies, such as those that produce most major LLMs, are naturally secretive about their systems' operations. However, this ‘black box’ opacity is untenable in scientific research. Thus, researchers must build systems that are transparent in their analyses and can be interrogated as to how they have drawn their conclusions.

## 6 TAKE‐HOMES

The integration of artificial intelligence into SR‐mTBI research represents a pivotal opportunity, offering tools of unprecedented power for understanding and mitigating injury, from designing novel experiments to identifying subtle patterns in complex data to personalising athlete rehabilitation. Yet, the risks of factual inaccuracy, algorithmic bias, and the erosion of scientific creativity demand a cautious and critical approach. The path forward must not be a blind embrace of technology, but a deliberate and ethical collaboration, where AI serves as a powerful instrument to complement, not replace, human intellect and accountability.

Thus, we as physiologists must work to innovate in the AI revolution, but with appropriate safeguards, spearheading the development of ethical and methodological frameworks to govern AI use in our field. This may take the form of developing large‐scale open‐source physiological databanks on which to train AIs while mitigating algorithmic bias, establishing shared AI model repositories to challenge the ‘black box’ issue of opacity, and defining rigorous standards for the use of generative AI. By harnessing its capabilities responsibly, the physiological community can ensure that this new chapter in neuroscience research leads to genuinely deeper understanding and, crucially, enhanced athlete welfare.

## AUTHOR CONTRIBUTIONS

Danny W. Walmsley and Damian M. Bailey conceived the idea and wrote the first draft of the manuscript. Danny W. Walmsley and Damian M. Bailey edited and revised the manuscript. Danny W. Walmsley and Damian M. Bailey approved the final version submitted for publication and agree to be accountable for all aspects of the work in ensuring that questions related to the accuracy or integrity of any part of the work are appropriately investigated and resolved. All persons designated as authors qualify for authorship, and all those who qualify for authorship are listed.

## CONFLICT OF INTEREST

D.M.B. is Editor‐in‐Chief of *Experimental Physiology*, Chair of the Life Sciences Working Group, member of the Human Spaceflight and Exploration Science Advisory Committee to the European Space Agency, member of the Space Exploration Advisory Committee to the UK and Swedish National Space Agencies and member of the National Cardiovascular Network for Wales and South‐East Wales Vascular Network. D.M.B. is also a consultant for Head for Change, a charity that provides care and support to those living with neurodegenerative diseases caused by their sporting careers.

## References

[eph70292-bib-0001] Antrobus, M. R. , Brazier, J. , Stebbings, G. K. , Day, S. H. , Heffernan, S. M. , Kilduff, L. P. , Erskine, R. M. , Williams, A. G. , & Pettitt, R. W. (2021). Genetic factors that could affect concussion risk in elite rugby. Sports, 9(2), 19. 33499151 10.3390/sports9020019 PMC7910946

[eph70292-bib-0002] Bailey, D. M. , Bain, A. R. , Hoiland, R. L. , Barak, O. F. , Drvis, I. , Hirtz, C. , Lehmann, S. , Marchi, N. , Janigro, D. , MacLeod, D. B. , Ainslie, P. N. , & Dujic, Z. (2022). Hypoxemia increases blood‐brain barrier permeability during extreme apnea in humans. Journal of Cerebral Blood Flow and Metabolism, 42(6), 1120–1135. 35061562 10.1177/0271678X221075967 PMC9121528

[eph70292-bib-0003] Beard, K. , Pennington, A. M. , Gauff, A. K. , Mitchell, K. , Smith, J. , & Marion, D. W. (2024). Potential Applications and Ethical Considerations for Artificial Intelligence in Traumatic Brain Injury Management. Biomedicines, 12(11), 2459. 39595025 10.3390/biomedicines12112459 PMC11592288

[eph70292-bib-0004] Ding, K. , Chetty, M. , Noori Hoshyar, A. , Bhattacharya, T. , & Klein, B. (2024). Speech based detection of Alzheimer's disease: A survey of AI techniques, datasets and challenges. Artificial Intelligence Review, 57(12), 325.

[eph70292-bib-0030] Fang, X. , Che, S. , Mao, M. , Zhang, H. , Zhao, M. , & Zhao, X. (2024). Bias of AI‐generated content: an examination of news produced by large language models. Scientific Reports, 14(5224), 2. 38433238 10.1038/s41598-024-55686-2 PMC10909834

[eph70292-bib-0005] Fang, Z. , Ran, H. , Zhang, Y. , Chen, C. , Lin, P. , Zhang, X. , & Wu, M. (2025). AlphaFold 3: An unprecedent opportunity for fundamental research and drug development. Precision Clinical Medicine, 8(3), pbaf015. 40799364 10.1093/pcmedi/pbaf015 PMC12342994

[eph70292-bib-0006] Janigro, D. , Bailey, D. M. , Lehmann, S. , Badaut, J. , O'Flynn, R. , Hirtz, C. , & Marchi, N. (2020). Peripheral Blood and Salivary Biomarkers of Blood–Brain Barrier Permeability and Neuronal Damage: Clinical and Applied Concepts. Frontiers in Neurology, 11, 577312. 33613412 10.3389/fneur.2020.577312 PMC7890078

[eph70292-bib-0007] Lui, Y. W. , Chang, P. D. , Zaharchuk, G. , Barboriak, D. P. , Flanders, A. E. , Wintermark, M. , Hess, C. P. , & Filippi, C. G. (2020). Artificial Intelligence in Neuroradiology: Current Status and Future Directions. AJNR. American Journal Of Neuroradiology, 41(8), E52–E59. 32732276 10.3174/ajnr.A6681 PMC7658873

[eph70292-bib-0008] Messeri, L. , & Crockett, M. J. (2024). Artificial intelligence and illusions of understanding in scientific research. Nature, 627(8002), 49–58. 38448693 10.1038/s41586-024-07146-0

[eph70292-bib-0009] Owens, T. S. , Calverley, T. A. , Stacey, B. S. , Iannatelli, A. , Venables, L. , Rose, G. , Fall, L. , Tsukamoto, H. , Berg, R. M. G. , Jones, G. L. , Marley, C. J. , & Bailey, D. M. (2021). Contact events in rugby union and the link to reduced cognition: Evidence for impaired redox‐regulation of cerebrovascular function. Experimental Physiology, 106(9), 1971–1980. 34355451 10.1113/EP089330

[eph70292-bib-0010] Owens, T. S. , Calverley, T. A. , Stacey, B. S. , Rose, G. , Fall, L. , Tsukamoto, H. , Jones, G. , Corkill, R. , Tuaillon, E. , Hirtz, C. , Marley, C. J. , & Bailey, D. M. (2021). Concussion history in rugby union players is associated with depressed cerebrovascular reactivity and cognition. Scandinavian Journal of Medicine and Science in Sports, 31(12), 2291–2299. 34487582 10.1111/sms.14046

[eph70292-bib-0011] Owens, T. S. , Marley, C. J. , Calverley, T. A. , Stacey, B. S. , Fall, L. , Tsukamoto, H. , Iannetelli, A. , Filipponi, T. , Davies, B. , Jones, G. L. , Marchi, N. , & Bailey, D. M. (2023). Lower systemic nitric oxide bioactivity, cerebral hypoperfusion and accelerated cognitive decline in formerly concussed retired rugby union players. Experimental Physiology, 108(8), 1029–1046. 37423736 10.1113/EP091195 PMC10988504

[eph70292-bib-0012] Patricios, J. S. , Schneider, K. J. , Dvorak, J. , Ahmed, O. H. , Blauwet, C. , Cantu, R. C. , Davis, G. A. , Echemendia, R. J. , Makdissi, M. , McNamee, M. , Broglio, S. , Emery, C. A. , Feddermann‐Demont, N. , Fuller, G. W. , Giza, C. C. , Guskiewicz, K. M. , Hainline, B. , Iverson, G. L. , Kutcher, J. S. , … Meeuwisse, W. (2023). Consensus statement on concussion in sport: The 6th International Conference on Concussion in Sport‐Amsterdam, October 2022. British Journal of Sports Medicine, 57(11), 695–711. 37316210 10.1136/bjsports-2023-106898

[eph70292-bib-0013] Reneker, J. C. , Babl, R. , & Flowers, M. M. (2019). History of concussion and risk of subsequent injury in athletes and service members: A systematic review and meta‐analysis. Musculoskeletal Science and Practice, 42, 173–185. 31014921 10.1016/j.msksp.2019.04.004

[eph70292-bib-0014] Rosenblatt, C. K. , Harriss, A. , Babul, A. N. , & Rosenblatt, S. A. (2021). Machine Learning for Subtyping Concussion Using a Clustering Approach. Frontiers in Human Neuroscience, 15, 716643. 34658816 10.3389/fnhum.2021.716643 PMC8514654

[eph70292-bib-0015] Salgado, S. , Saver, V. , Sáenz, Á. , Ferre, A. , Giglio, A. , & Reccius, A. (2026). Biomarkers and clinical rules for the management of mild traumatic brain injury: A narrative review. International Journal of Emergency Medicine, 19(1), 24. 41580617 10.1186/s12245-025-01088-8 PMC12833935

[eph70292-bib-0016] Sparkes, A. , Aubrey, W. , Byrne, E. , Clare, A. , Khan, M. N. , Liakata, M. , Markham, M. , Rowland, J. , Soldatova, L. N. , Whelan, K. E. , Young, M. , & King, R. D. (2010). Open Access REVIEW Towards Robot Scientists for autonomous scientific discovery. Automated Experimentation, 2, 1. 20119518 10.1186/1759-4499-2-1 PMC2813846

[eph70292-bib-0017] Szymanski, N. J. , Rendy, B. , Fei, Y. , Kumar, R. E. , He, T. , Milsted, D. , McDermott, M. J. , Gallant, M. , Cubuk, E. D. , Merchant, A. , Kim, H. , Jain, A. , Bartel, C. J. , Persson, K. , Zeng, Y. , & Ceder, G. (2023). An autonomous laboratory for the accelerated synthesis of novel materials. Nature, 624(7990), 86–91. 38030721 10.1038/s41586-023-06734-w PMC10700133

[eph70292-bib-0018] Thomas, G. A. , & Arnett, P. A. (2025). Get Your Brain in the Game: Using Machine Learning to Predict Recovery Timelines Following Sports‐Related Concussion. Archives of Clinical Neuropsychology, 40(8), 1533–1545. 40685569 10.1093/arclin/acaf066

[eph70292-bib-0019] Tom, G. , Schmid, S. P. , Baird, S. G. , Cao, Y. , Darvish, K. , Hao, H. , Lo, S. , Pablo‐García, S. , Rajaonson, E. M. , Skreta, M. , Yoshikawa, N. , Corapi, S. , Akkoc, G. D. , Strieth‐Kalthoff, F. , Seifrid, M. , & Aspuru‐Guzik, A. (2024). Self‐Driving Laboratories for Chemistry and Materials Science. Chemical Reviews, 124(16), 9633–9732. 39137296 10.1021/acs.chemrev.4c00055 PMC11363023

[eph70292-bib-0020] Varadi, M. , Anyango, S. , Deshpande, M. , Nair, S. , Natassia, C. , Yordanova, G. , Yuan, D. , Stroe, O. , Wood, G. , Laydon, A. , Zídek, A. , Green, T. , Tunyasuvunakool, K. , Petersen, S. , Jumper, J. , Clancy, E. , Green, R. , Vora, A. , Lutfi, M. , … Velankar, S. (2022). AlphaFold Protein Structure Database: Massively expanding the structural coverage of protein‐sequence space with high‐accuracy models. Nucleic Acids Research, 50(D1), D439–D444. 34791371 10.1093/nar/gkab1061 PMC8728224

[eph70292-bib-0021] Wagner, G. , Lukyanenko, R. , & Paré, G. (2022). Artificial intelligence and the conduct of literature reviews. Journal of Information Technology, 37(2), 209–226.

[eph70292-bib-0022] Wilde, E. A. , Wanner, I. B. , Kenney, K. , Gill, J. , Stone, J. R. , Disner, S. , Schnakers, C. , Meyer, R. , Prager, E. M. , Haas, M. , & Jeromin, A. (2022). A Framework to Advance Biomarker Development in the Diagnosis, Outcome Prediction, and Treatment of Traumatic Brain Injury. Journal of Neurotrauma, 39(7–8), 436–457. 35057637 10.1089/neu.2021.0099 PMC8978568

[eph70292-bib-0023] Woo, P. Y. M. , Cheung, E. , Lau, F. W. Y. , Law, N. W. S. , Mak, C. K. Y. , Tan, P. , Siu, B. , Wong, A. , Mak, C. H. K. , Chan, K. Y. , Yam, K. Y. , Pang, K. Y. , Po, Y. C. , Lui, W. M. , Chan, D. T. M. , & Poon, W. S. (2021). Multicentre study of hospitalised patients with sports‐and recreational cycling–related traumatic brain injury in hong kong. Hong Kong Medical Journal, 27(5), 338–349. 34706984 10.12809/hkmj208934

[eph70292-bib-0025] Zhan, X. , Liu, Y. , Cecchi, N. J. , Towns, J. , Callan, A. A. , Gevaert, O. , Zeineh, M. M. , & Camarillo, D. B. (2026). Identification of head impact locations, speeds, and force based on head kinematics. IEEE Transactions on Biomedical Engineering, 73, 293–300. 40536866 10.1109/TBME.2025.3581171

